# Systematic Review and Meta-Analysis of In Vitro Anti-Human Cancer Experiments Investigating the Use of 5-Aminolevulinic Acid (5-ALA) for Photodynamic Therapy

**DOI:** 10.3390/ph14030229

**Published:** 2021-03-07

**Authors:** Yo Shinoda, Daitetsu Kato, Ryosuke Ando, Hikaru Endo, Tsutomu Takahashi, Yayoi Tsuneoka, Yasuyuki Fujiwara

**Affiliations:** Department of Environmental Health, School of Pharmacy, Tokyo University of Pharmacy and Life Sciences, 1432-1 Horinouchi, Hachioji, Tokyo 192-0392, Japan; y151041@toyaku.ac.jp (D.K.); y151008@toyaku.ac.jp (R.A.); doendoenshning1403@gmail.com (H.E.); tsutomu@toyaku.ac.jp (T.T.); tsuneoka@toyaku.ac.jp (Y.T.)

**Keywords:** 5-aminolevulinic acid, 5-ALA, dALA, δALA, PpIX, protoporphyrin IX, photodynamic therapy, PDT

## Abstract

5-Aminolevulinic acid (5-ALA) is an amino acid derivative and a precursor of protoporphyrin IX (PpIX). The photophysical feature of PpIX is clinically used in photodynamic diagnosis (PDD) and photodynamic therapy (PDT). These clinical applications are potentially based on in vitro cell culture experiments. Thus, conducting a systematic review and meta-analysis of in vitro 5-ALA PDT experiments is meaningful and may provide opportunities to consider future perspectives in this field. We conducted a systematic literature search in PubMed to summarize the in vitro 5-ALA PDT experiments and calculated the effectiveness of 5-ALA PDT for several cancer cell types. In total, 412 articles were identified, and 77 were extracted based on our inclusion criteria. The calculated effectiveness of 5-ALA PDT was statistically analyzed, which revealed a tendency of cancer-classification-dependent sensitivity to 5-ALA PDT, and stomach cancer was significantly more sensitive to 5-ALA PDT compared with cancers of different origins. Based on our analysis, we suggest a standardized in vitro experimental protocol for 5-ALA PDT.

## 1. Introduction

5-Aminolevulinic acid (5-ALA) is a naturally occurring amino acid derivative that acts as a precursor of protoporphyrin IX (PpIX) [1,2,3]. 5-ALA administration to animals, including humans, leads to the synthesis of PpIX, especially in tumors [4,5,6,7]. PpIX is activated by violet light (405 nm) or orange-red light (635 nm), subsequently emitting red fluorescence (620–710 nm) or generating reactive oxygen species (ROS) [8]. These features can potentially be used to visualize or kill cancer. Specifically, 5-ALA has been clinically tested for photodynamic diagnosis (PDD) during surgery to visualize cancer cells by fluorescence and photodynamic therapy (PDT) to target unfavorable neoplasms by increasing ROS production [9,10]. To date, 5-ALA has been clinically approved by the U.S. Food and Drug Administration (FDA) as GLEOLAN^®^ (GLIOLAN^®^ according to the European Medicines Agency (EMA)) for PDD for malignant glioma, and LEVULAN^®^ and AMELUZ^®^ have been approved for the PDT of patients with actinic keratoses. However, there are no FDA- or EMA-approved applications of 5-ALA-PDT for cancer.

Clinical anti-cancer applications of 5-ALA-PDT have been widely reported for several organs, such as the brain [11,12,13,14,15,16], skin [17,18,19,20,21,22,23], pharynx [24], blood and lymph [25], esophagus [26], urethra and prostate [27], and uterus [28]. In addition, 97 clinical trials of 5-ALA PDT for cancer treatment have been registered in the U.S. National Library of Medicine (ClinicalTrials.gov) as of 10 March 2021. Therefore, 5-ALA will hopefully be approved in the near future as a PDT drug for cancer patients. These clinical trials and applications are based on in vivo animal experiments, and these animal experiments are based on in vitro cell culture experiments. For the clinical application of 5-ALA PDT in cancer, a comprehensive review of in vitro 5-ALA PDT experiments and analysis of these results are meaningful and may provide important opportunities to consider for the future direction of 5-ALA experiments and clinical trials.

In this study, we systematically extracted and listed in vitro experiments that investigated 5-ALA PDT. We also performed a meta-analysis of these data by calculating and comparing the effectiveness of 5-ALA PDT in several cancer cell types from each article. Finally, we suggest a standard experimental protocol for the validation of future in vitro 5-ALA PDT experiments.

## 2. Methods

### 2.1. Literature Search and Selection

A systematic literature review using PubMed was performed according to the Preferred Reporting Items for Systematic Reviews and Meta-analyses Statement (PRISMA) guidelines [29]. For the title or abstract search, we used the term sets [5-aminolevulinic acid, aminolevulinic acid, dALA, δALA, 5-ALA, 5ALA], [in vitro, culture], and [photodynamic therapy, PDT] for OR searching in each term set, and each term set was used together for AND searching. In addition, the publication date was limited to the beginning of 1900 to the end of 2019 (available online). Together, the search query used was ((aminolevulinic acid [Title/Abstract] OR aminolevulinic acid [Title/Abstract] OR dALA [Title/Abstract] OR δALA [Title/Abstract] OR 5-ALA [Title/Abstract] OR 5ALA [Title/Abstract]) AND (in vitro [Title/Abstract] OR culture [Title/Abstract]) AND (photodynamic therapy [Title/Abstract] OR PDT [Title/Abstract])) AND (1900/01/01 [Date-Publication]: 2019/12/31 [Date-Publication]). The searched articles were further selected based on whether they included all of the following information: cell name, fluence, irradiation wavelength, time of incubation with 5-ALA, duration between 5-ALA treatment and irradiation, and duration between irradiation and viability assays. The selected articles that described the median lethal concentration (LC_50_) in the text or those in which the LC_50_ could be estimated and/or calculated from the table or graph were included. Estimated LC_50_ and fluence from graphs were rounded.

### 2.2. Consistency of Terminology

Some papers used different terms to express the same thing. In addition, some standardizations of different terms were required to perform statistical analysis. Therefore, some terminologies were unified as follows: astrocytoma was used as glioblastoma, glioma stem-like cell was used as glioma stem cell, and glioblastoma stem-like cell was used as glioblastoma stem cell.

### 2.3. Data Collection, Processing, and Statistics

For data comparisons, the effectiveness of each application was calculated by the reciprocal of the fluence multiplied by the LC_50_ (cm^2^/(J·µM)). This effectiveness is thought to be proportional to the sensitivity of the cell to 5-ALA PDT. If there were more than three articles that used the same classified cells or cells from the same organ, similar wavelengths for irradiation (around 635 nm), the same duration of 5-ALA incubation (4 h), and the same duration between irradiation and viability assays (24 h), the values of effectiveness were averaged and assessed by one-way analysis of variance (ANOVA) with the post hoc Tukey–Kramer test using Statcel2 software (Seiunsha, Tokyo, Japan). If the sample size was greater than six, the data were assessed by the Wilcoxon rank-sum test using JMP Pro software (SAS Institute Japan, Tokyo, Japan).

## 3. Results

### 3.1. Collection of In Vitro 5-ALA PDT Experiments

The initial search resulted in 412 articles, of which 77 articles met the inclusion criteria mentioned in the Methods section. These articles included a total of 146 in vitro viability assays of cells treated with PDT under different conditions. They included 116, 12, 9, and 9 viability assays for cell lines derived from humans, mice, rats, and canines, respectively. The PDT experiments using human cell lines are listed in Table 1, and the total number of studies was 62. Eighty cell lines from different origins (21 organs) and classifications (16 classes) were tested. Brain cancer and adenocarcinoma were the most tested cancer origin and classification, respectively. Overall, several different experimental conditions were adapted in each study, including various durations of incubation with 5-ALA, irradiation wavelengths, fluences, and durations between irradiation and viability assays. Therefore, it was difficult to directly compare these experimental results. To ensure the comparability of the results, we extracted the data from studies that used similar experimental conditions (irradiation wavelength, duration of 5-ALA incubation, and duration between irradiation and viability assays). Then, we roughly estimated the effectiveness of 5-ALA PDT for different cells using the following equation. The effectiveness is the new parameter we introduced, which is thought to correlate to the sensitivity of the cells to 5-ALA PDT because both LC_50_ and fluence parameters are roughly inversely proportional to the sensitivity of the cells in PDT experiments [30,31].

(1) $$Effectiveness=\frac{1}{{LC}_{50}\times Fluence}\left({cm}^{2}/\left[J\cdot µM\right]\right)$$

The effectiveness indicates the extent of 5-ALA effects on the treated cells under the individual experimental conditions. The larger the effectiveness value, the more effective 5-ALA PDT was against the cell. Although there were different effectiveness values estimated for the same cell types in different papers (such as 0.1 to 1,131.9 for A431 cells), most results showed a similar range of effectiveness values.

### 3.2. 5-ALA PDT Effect on Cells of Different Cancer Classifications

**Figure 1:**
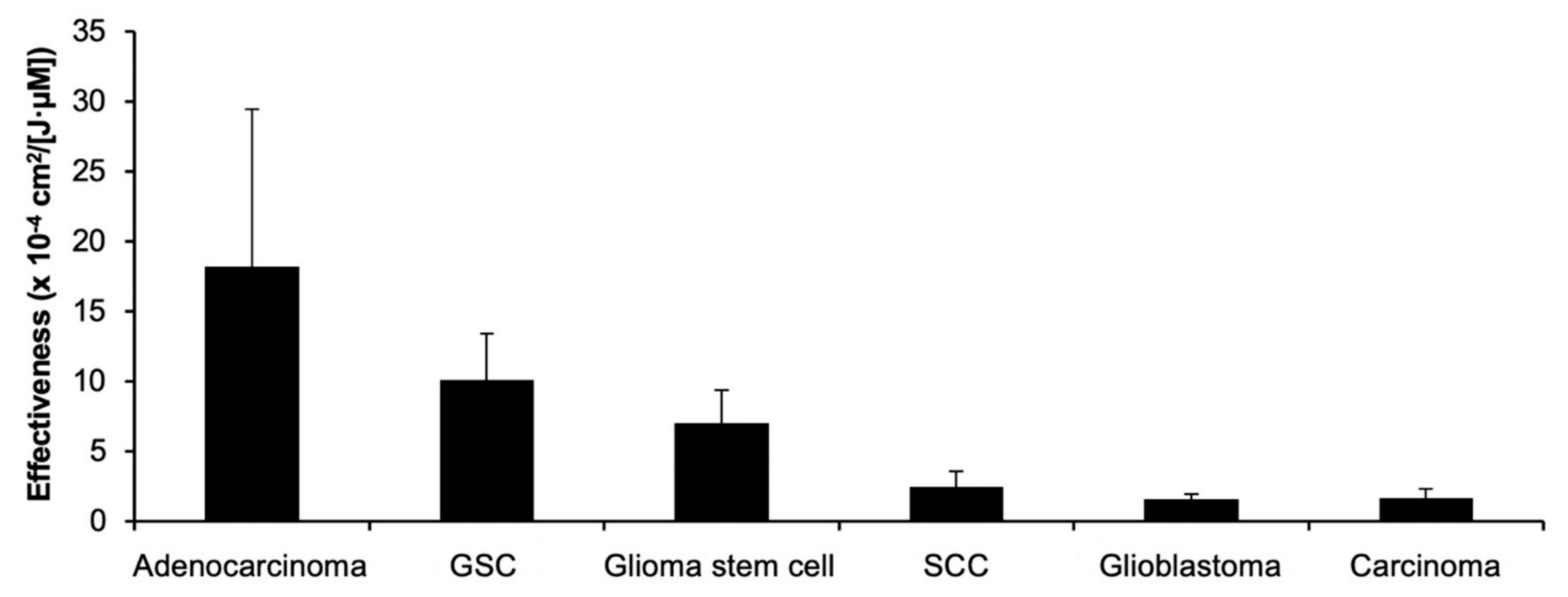
Effectiveness of 5-ALA PDT in cells of different cancer classifications. GSC: glioblastoma stem cell, SCC: squamous cell carcinoma. There were no statistical significances (one-way analysis of variance (ANOVA) with the post hoc Tukey–Kramer test). Adenocarcinoma (*n* = 16) and glioblastoma (*n* = 10) were statistically assessed using the Wilcoxon rank-sum test but showed no significant differences.

The effectiveness against cells of the same cancer classifications was averaged and compared with each class (Figure 1). Several reports showed that the feature of the cells was altered by their microenvironments, such as 2D monolayer culture or 3D aggregation-forming spheres [90,91,92,93]. In the present review, because the inclusion and exclusion of the data from sphere cultures did not show any statistical differences, all of the data were included and averaged. Although there were no statistical significances, adenocarcinoma, glioblastoma stem cell (GSC), and glioma stem cell tended to show higher effectiveness values than squamous cell carcinoma (SCC), glioblastoma, and carcinoma, which may suggest that the effects of 5-ALA PDT are cancer-classification-dependent.

### 3.3. 5-ALA PDT Effect on Cells of Different Cancer Origins

**Figure 2:**
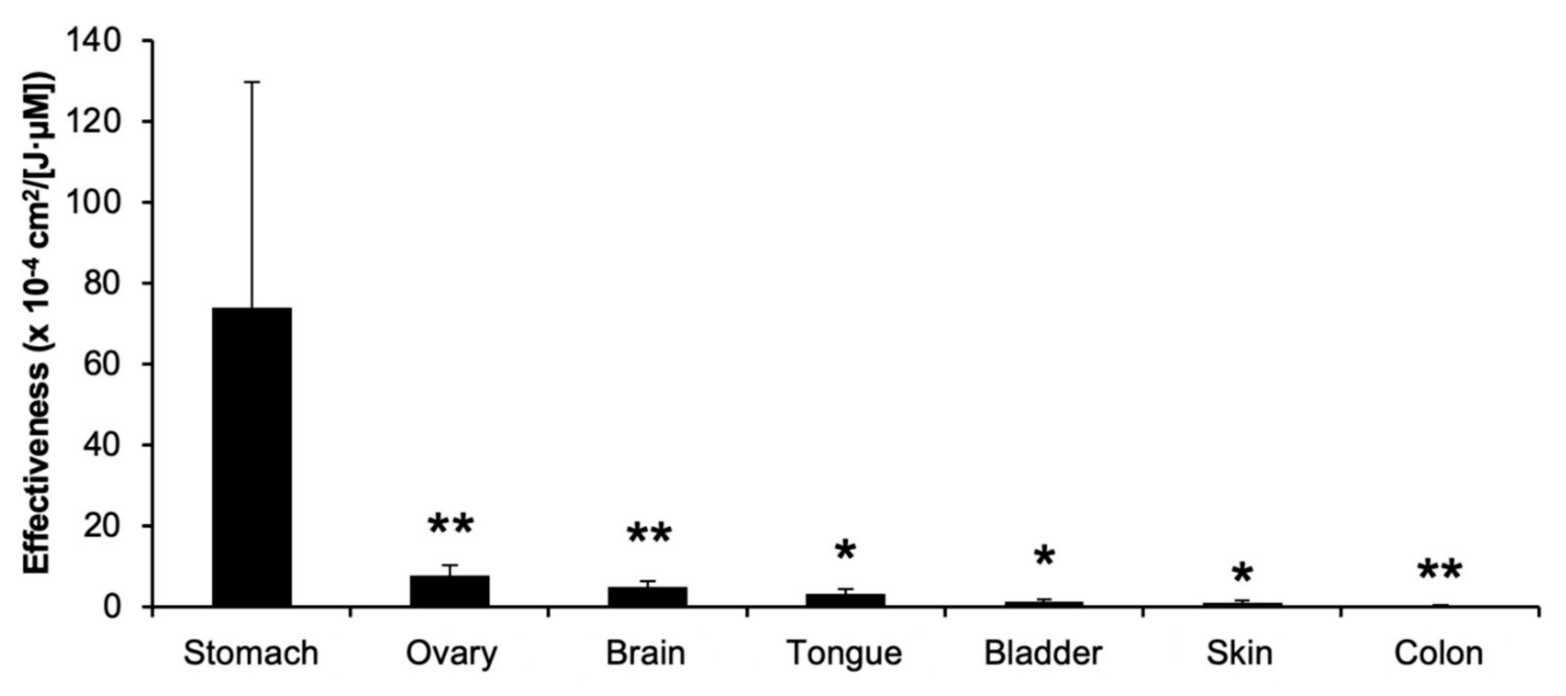
Effectiveness of 5-ALA PDT on cells of different cancer origins. The stomach was identified as the organ most affected by 5-ALA PDT (one-way analysis of variance (ANOVA) with the post hoc Tukey–Kramer test). * *p* < 0.05 and ** *p* < 0.01 compared with the stomach. The brain (*n* = 18) and ovary (*n* = 7) were statistically assessed using the Wilcoxon rank-sum test but showed no significant differences.

Next, the effectiveness against cells of the same cancer origin was averaged and compared with each origin (Figure 2). Because the inclusion and exclusion of the data from sphere cultures and outliers did not show any statistical differences, all of the data were included and averaged. As a result, the stomach was identified as the organ most affected by 5-ALA PDT. Among the other organs, there were no statistically significant differences. However, the number of experiments using stomach-derived cells (*n* = 3) was small, and these experiments were performed in the same study. Therefore, this result should be considered carefully.

## 4. Discussion and Future Perspective

In the present review, we summarized past and recent (until the end of 2019) in vitro experiments investigating 5-ALA PDT for cancer cells and compared these data by calculating the effectiveness value. In total, 116 in vitro assays for human cancer cells were extracted, including cancer cells from 21 origins and 16 cancer classifications. Effectiveness values were calculated from the LC_50_ and fluence to compare the sensitivity of each cancer cell type to 5-ALA PDT. These data suggest that there are some tendencies of sensitivity to 5-ALA PDT in cells of different origins and classifications.

Several potential mechanisms may contribute to the differences in the sensitivity of each origin and classification to 5-ALA PDT. The most important candidate that influences the PDT sensitivity is the protein family associated with redox reactions. Oxidative stress-related proteins, such as superoxide dismutase [94], catalase [95], and NO synthase [96], are reported to affect PDT sensitivity. Glutathione and related proteins, such as glutathione peroxidase, glutathione-S-transferase, glutathione transferase omega-1, and glutathione synthase, are also thought to be associated with cell resistance to PDT [94,97,98]. Heme oxygenase-1 (HO-1) is an inducible cytoprotective enzyme that protects cells from oxidative stress, and its expression is induced by PDT [99]. Apurinic/apyrimidinic endonuclease 1/redox factor-1 (APE1/Ref-1) regulates cell responses to oxidative stress, which affects the PDT sensitivity [100]. The NAD(P)H/FAD redox status has been reported to affect PDT sensitivity [101]. The expression levels of these redox-related proteins, peptides, and compounds can be altered in cancer cells and may influence their sensitivity to 5-ALA PDT.

Several papers have reported the anti-cancer property of 5-ALA PDT; however, most articles do not describe all of the experimental protocols, preventing reproducibility. Although similar problems occur and should be considered in all manuscripts, it is still important for authors to describe their detailed experimental protocols to ensure reproducibility by other researchers and for reviewers to carefully assess the manuscripts. For the in vitro PDT experiments, complete chemical formulation of 5-ALA (such as 5-ALA hydrochloride) should be described, not 5-ALA alone; otherwise, the dimerization is reported [102]. Moreover, the parameters we mention in Table 1 (cell name, duration of incubation, irradiation wavelength, fluence, and duration between irradiation and viability assays) should be clearly mentioned in Section 2 because all of these parameters possibly affect the sensitivity of cells to 5-ALA PDT and the results of cell viability assays. Comparisons of the duration of 5-ALA incubation [35,68,103], irradiation wavelength [104,105], and fluence [30,31] showed that each parameter strongly affected cell viability. In addition, the duration between irradiation and viability assays can also affect the results because cell proliferation after light irradiation can be influenced by the number of viable cells with a sigmoid shape.

In this review, we compared the effectiveness calculated from the LC_50_ and fluence. The calculated effectiveness may be a useful parameter to compare the sensitivity of cells to 5-ALA PDT among individual manuscripts; however, it has some limitations to consider. Although both LC_50_ and fluence parameters are roughly inversely proportional to the sensitivity of the cells in PDT experiments, this inverse relationship (especially for fluence) does not show absolute linearity [31]. Based on our calculation, high fluence usually showed a relatively high effectiveness [30,57,106,107]. Therefore, the calculated effectiveness may be overestimated in the experiments using high fluence. The cellular microenvironments, such as 2D or 3D culture, are also possible candidates that may affect 5-ALA PDT sensitivity. Therefore, 2D/3D comparisons might be required. In this manuscript, the inclusion and exclusion of 3D culture data did not show any statistical changes, but this might be caused by the small number of datasets. We discarded several potential parameters that may affect in vitro PDT assays, such as the components of 5-ALA (hydrochloric acid, nitrate, and phosphate), initial cell density, light source (laser, lamp (halogen, mercury, or xenon), or LED), light irradiance, wash conditions, and completeness of light shielding except for light irradiation. These parameters can also affect the PDT results. For example, the value of light irradiance strongly influences cell viability [105,108,109]. Therefore, especially for the reproducibility, these parameters should be described in the individual manuscript together with the parameters described above.

**Scheme 1:**
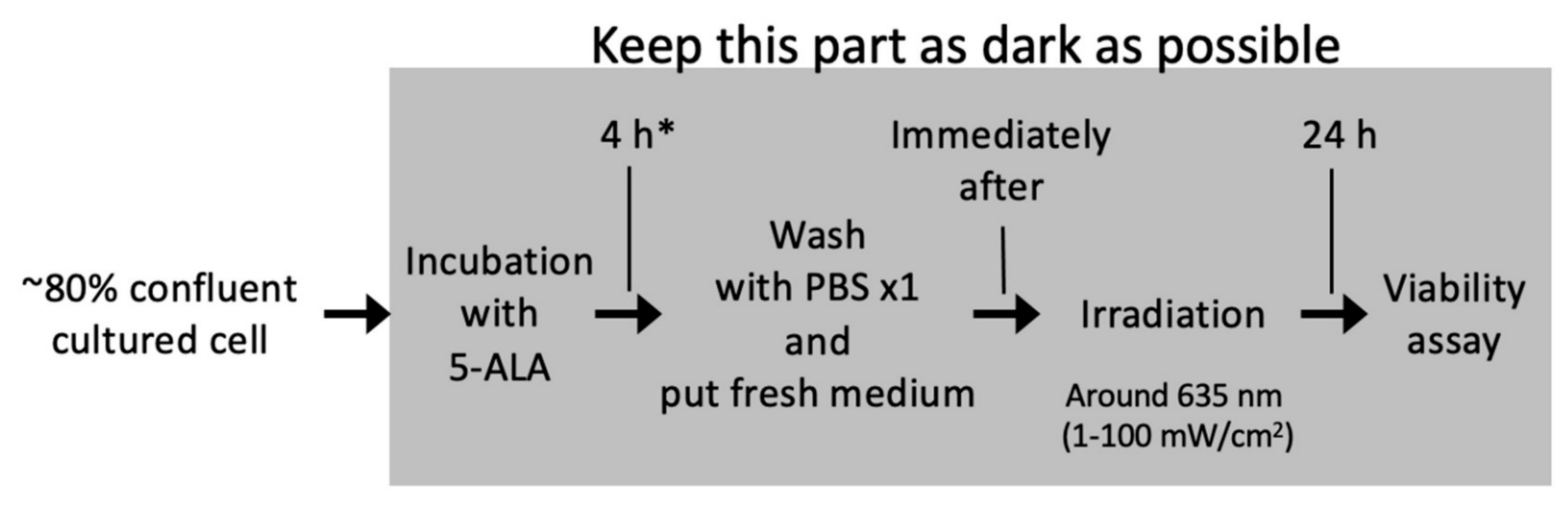
Recommended experimental protocol for 5-ALA PDT. This protocol can be used as a standard protocol for in vitro 5-ALA PDT experiments. The duration of incubation (4 h may be a standard) can be changed if incubation time-dependency is investigated (*). Irradiance is particularly difficult to adjust because the light source is different in each lab, but the recommended irradiance is around 1 to 100 mW/cm^2^. Note that the experimental procedure indicated inside the gray box should be performed in the dark as much as possible because undesirable irradiation from the fluorescent lights of laminar flow cabinets and/or experimental rooms can increase ROS production and subsequent cell death. PBS: phosphate-buffered saline.

For the meta-analysis and data comparison between each report, the experimental procedure for in vitro 5-ALA PDT experiments should be standardized as much as possible. We propose a standardized protocol developed with reference to papers listed in Table 1 (Scheme 1), and a similar protocol for investigating 5-ALA PDT in breast cancer cells has been recommended previously [110]. This protocol is for in vitro 5-ALA PDT experiments, but it can potentially be used for other in vitro PDT experiments using different photosensitizers with some modifications. The steps of this protocol are culturing cells to ~80% confluency, incubating cells with 5-ALA for 4 h, irradiating immediately after providing cells with fresh medium, and performing the viability assay 24 h after irradiation. A cell confluency of approximately 80% should be used for prevention of the effect of contact inhibition (including initiation of cell cycle arrest, downregulation of proliferation, and mitogen signaling pathways) [111]. It has been reported that a 4 h incubation time of 5-ALA is usually sufficient to induce maximal PDT effect for several cancer cells [60,65,112]. In addition, many studies including the references in this review used 4 h for their general experimental condition. However, some cells and experimental conditions require the incubation time of 5-ALA to be more than 4 h [62,85,107,113] and the time may also be affected by the 5-ALA concentration [35]. The timing of irradiation should be performed immediately after washout of 5-ALA because intracellular concentration of PpIX is gradually reduced after washout by cell metabolism [114]. We hope that this standardized protocol may help researchers conducting 5-ALA (or other photosensitizer) PDT experiments.

More in vitro and in vivo 5-ALA PDT experiments for cancer should be performed to facilitate the clinical application of 5-ALA PDT in the future. In addition, 81 clinical trials have been registered in the U.S. National Library of Medicine. There are potential risks and side effects that should be considered during these trials and experiments; however, these efforts might advance the development of novel clinical approaches for the treatment of several cancers. To expand 5-ALA applications for several cancers, further in vitro 5-ALA PDT experiments are still required and should be continued.

## Figures and Tables

**Table 1:** In vitro 5-Aminolevulinic acid photodynamic therapy (5-ALA PDT) experiments for human cancer cell lines.

Organ	Classification	Name [s]: Sphere	Effectiveness (×10^−4^ cm^2^/(J·µM))	LC_50_ (μM)	Duration of Incubation (h)	Irradiation Wavelength (nm)	Fluence (J/cm^2^)	Duration between Irradiation and Viability Assay (h)	Ref.
**Bladder**	Carcinoma	HCV-29	0.2	597	4	635	100	24	[32]
	Carcinoma	J82	1.1	597	4	635	15	24	[32]
	Carcinoma	J82	12.0	597	3	590–700	1.4	48	[33]
	Carcinoma	RT112	41.9	597	3	590–700	0.4	48	[33]
	Carcinoma	RT4	2.2	298	4	635	15	24	[32]
	Carcinoma	RT4	41.9	597	3	590–700	0.4	48	[33]
	Carcinoma	RT4 [s]	20.9	597	3	400–700	0.8	24	[34]
**Bone**	Chordoma	U-CH2	3.0	181	6	635	18.75	24	[35]
	Osteosarcoma	HOSM-1	2.5	200	6	580–740	20	24	[36]
	Osteosarcoma	HOSM-2	0.5	1000	12	600–1600	20	24	[37]
**Brain**	AT/RT	BT-16	1.1	370	4	635	25	12	[38]
	Glioblastoma	A172	3.3	1000	24	635	3	24	[39]
	Glioblastoma	ACBT [s]	0.6	597	4	635	30	24	[40]
	Glioblastoma	U251MG	3.3	1000	0.5	All (white)	3	20-24	[41]
	Glioblastoma	U251MG	5.0	1000	4	627	2	O/N	[42]
	Glioblastoma	U373	3.7	144	4	635	18.8	24	[43]
	Glioblastoma	U373	1.2	650	4	635	12.75	24	[44]
	Glioblastoma	U373	3.2	315	4	635 ± 20	10	24	[45]
	Glioblastoma	U373MG	3.5	500	2	635	5.7	48	[46]
	Glioblastoma	U373MG	7.1	1000	4	627	1.4	O/N	[47]
	Glioblastoma	U373MG	5.1	1000	4	635	1.95	48	[48]
	Glioblastoma	U373vIII	0.7	1100	4	635	12.75	24	[44]
	Glioblastoma	U373vIII	2.5	407	4	635 ± 20	10	24	[45]
	Glioblastoma	U87	1.5	510	4	635	12.75	24	[44]
	Glioblastoma	U87	1.1	931	4	635 ± 20	10	24	[45]
	Glioblastoma	U87MG	2.5	1000	0.5	All (white)	4	20–24	[41]
	Glioblastoma	U87MG	2.4	1000	6	634 ± 7	4.1	48	[31]
	Glioblastoma	U87MG	4.2	1000	4	627	2.4	O/N	[47]
	Glioblastoma	U87MG	3.3	1000	4	627	3	O/N	[42]
	Glioblastoma	U87vIII	0.3	2800	4	635	12.75	24	[44]
	Glioblastoma	U87vIII	0.9	1161	4	635 ± 20	10	24	[45]
	GSC	BT273 [s]	4.4	122	4	635	18.8	24	[43]
	GSC	BT275 [s]	10.7	49.5	4	635	18.8	24	[43]
	GSC	BT379 [s]	8.8	60.3	4	635	18.8	24	[43]
	GSC	GS3 [s]	4.3	124	4	635	18.8	24	[43]
	GSC	GS5 [s]	22.3	23.9	4	635	18.8	24	[43]
	Glioma stem cell	GS2	2.6	298	4	635	12.75	24	[44]
	Glioma stem cell	GS2	7.7	130	4	635 ± 20	10	24	[45]
	Glioma stem cell	GSC30 [s]	10.8	93	4	635 ± 20	10	24	[45]
	Medulloblastoma	D283 Med	5.3	500	2	635	3.8	48	[46]
	Medulloblastoma	Daoy	1.7	239	4	635	25	12	[38]
	Meningioma	KT21-MG1	1.2	448	24	635	18.75	1.5	[49]
	Neuroblastoma	SK-N-SH	1.4	1000	8	500–	7.2	48	[50]
	PNET	PFSK-1	1.7	239	4	635	25	12	[38]
**Breast**	Adenocarcinoma	MDA-MB-231	5.0	1000	0.5	633 ± 6	2	20	[51]
	Adenocarcinoma	MDA-MB-231	4.3	1000	6	634 ± 7	2.3	48	[31]
	Carcinoma	HB4a-Ras	166.7	1000	3	400-700	0.06	19	[52]
	Carcinoma	T47D	5.0	500	24	624 ± 5	4	24	[53]
	Carcinoma	T47D	16.7	1000	4	635	0.6	48	[48]
**Colon**	Adenocarcinoma	Caco-2	9.9	597	3	590–700	1.7	48	[54]
	Adenocarcinoma	HT-29	2.5	1000	3	635	4	24	[55]
	Adenocarcinoma	HT-29	4.3	597	3	590–700	3.9	48	[54]
	Adenocarcinoma	SW480	0.1	1500	4	600–720	50	24	[56]
	Adenocarcinoma	SW480	0.1	1500	4	600–720	53	24	[57]
	Adenocarcinoma	SW480	4.9	597	3	590–700	3.4	48	[54]
	Adenocarcinoma	SW620	0.4	1500	4	600–720	18	24	[56]
	Adenocarcinoma	SW620	0.4	1000	4	600–720	24	24	[57]
**Esophagus**	SCC	Eca-109	1.0	1000	24	630	10	24	[58]
	SCC	Eca-109	0.1	750	6	630	100	24	[59]
**Gingiva**	SCC	Ca9-22	20.8	1000	3	633	0.48	24	[60]
**Hypopharynx**	SCC	FADU	8.9	1000	24	635	1.12	24	[39]
**Kidney**	Carcinoma	A498	1.2	1000	6	634 ± 7	8.2	48	[31]
**Larynx**	SCC	AMC-HN3	7.0	239	24	632	6	24	[61]
**Liver**	Carcinoma	HepG2	10.0	1000	28	600–800	1	2	[62]
	Carcinoma	HepG2	2.2	185	3	632	25	24	[63]
**Lung**	Adenocarcinoma	LC-T	1.0	5000	9	600–700	2.1	0	[64]
	Carcinoma	H1299	5.0	1000	4	633	2	2.3	[65]
	Carcinoma	QU-DB	0.8	5000	9	600–700	2.5	0	[64]
**Lymph**	Lymphoma	HuT78	41.9	59.7	2	630	4	24	[66]
	Lymphoma	Ramos (RA1)	16.8	59.7	2	630	10	24	[66]
**Nasopharynx**	Carcinoma	HNE-1	3.0	328	4	630	10	24	[67]
	Carcinoma	KJ-1	3.6	1000	3	633	2.8	24	[60]
**Oral Cavity**	Dysplasia	DOK	1.2	810	4	635	10	24	[68]
**Ovary**	Adenocarcinoma	ES2	1.1	882	4	631	10.4	24	[69]
	Adenocarcinoma	KOC7C	1.1	857	4	631	10.4	24	[69]
	Adenocarcinoma	OV2774	1.3	1000	4	635	8	48	[48]
	Adenocarcinoma	OVMANA	9.9	97	4	631	10.4	24	[69]
	Adenocarcinoma	OVTOKO	3.9	244	4	631	10.4	24	[69]
	Adenocarcinoma	RMG1	17.1	56	4	631	10.4	24	[69]
	Adenocarcinoma	RMG2	17.1	56	4	631	10.4	24	[69]
	Adenocarcinoma	TOV21G	2.9	330	4	631	10.4	24	[69]
**Prostate**	Adenocarcinoma	LNCaP	11.2	298	4	631	3	24	[70]
**Skin**	Melanoma	A375	2.0	500	4	636	10	24	[71]
	Melanoma	A375	0.6	358	4	420–1400	45	24	[72]
	Melanoma	LOX	0.03	4000	4	635	100	20	[73]
	SCC	A431	0.6	6000	3	635 ± 9	3	0	[74]
	SCC	A431	17.0	393	20	630	1.5	24	[30]
	SCC	A431	12.5	100	24	632.8	8	24	[75]
	SCC	A431	0.1	2000	4	635	40	24	[76]
	SCC	A431	1131.9	1.77	48	630 ± 15	5	48	[77]
	SCC	HSC-5	1.0	200	2	545–700	50	3	[78]
	SCC	SCC-13	0.1	6000	1	635 ± 9	12.21	0	[74]
**Stomach**	Adenocarcinoma	KKLS	13.2	700	4	630	1.08	24	[79]
	Adenocarcinoma	MKN28	23.1	400	4	630	1.08	24	[79]
	Adenocarcinoma	MKN45	185.2	50	4	630	1.08	24	[79]
**Tongue**	SCC	CAL-27	1.6	620	4	635	10	24	[68]
	SCC	SCC-15	11.2	59.7	12	630	15	6	[80]
	SCC	SCC-4	5.3	187	4	640	10	24	[81]
	SCC	SCC-4	2.7	375	4	640	10	24	[82]
**Uterus**	Adenocarcinoma	BCC	16.7	500	4.5	532 ± 20	1.2	20	[83]
	Adenocarcinoma	HeLa	4.0	500	4	635	5	24	[84]
	Adenocarcinoma	HeLa	100.0	200	8	630	0.5	24	[85]
	Adenocarcinoma	HeLa	98.0	10.2	6	630	10	3	[86]
	Adenocarcinoma	HeLa	0.3	300	4	635	100	20	[87]
	Adenocarcinoma	HeLa	16.7	1000	24	635	0.6	24	[88]
	Adenocarcinoma	KB	1.3	200	6	580–740	40	24	[36]
	SCC	C-33A	588.2	1.7	6	630	10	3	[86]
	SCC	C-4 I	12.9	77.7	6	630	10	3	[86]
	SCC	Ca Ski	28.4	35.2	6	630	10	3	[86]
	SCC	HT-3	3.0	332	6	630	10	3	[86]
	SCC	Me-180	1373.6	0.728	6	630	10	3	[86]
	SCC	Me-180	660.1	0.505	4	632.8	30	4	[89]
	SCC	SiHa	3.0	332	6	630	10	3	[86]

AT/RT: atypical teratoid/rhabdoid tumor, GSC: glioblastoma stem cell, SCC: squamous cell carcinoma, PNET: primitive neuroectodermal tumor, O/N: overnight; LC_50_, median lethal concentration.
